# Transient Receptor Potential Ankyrin 1 and Vanilloid 1 Receptor mRNAs Are Not Translated to Functional Ion Channels in Human Prostate Adenocarcinoma Cells

**DOI:** 10.3390/cells15211986

**Published:** 2026-10-30

**Authors:** Anita Steib, Katalin Rozmer, Zoltán Sándor, Éva Szőke, Dávid Semjén, Béla Kajtár, Krisztina Pohóczky, Zsuzsanna Helyes

**Affiliations:** 1 Department of Pharmacology and Pharmacotherapy, Medical School, University of Pécs, 7624 Pécs, Hungary; steib.anita@pte.hu (A.S.); kovacs-rozmer.katalin@pte.hu (K.R.); zoltan.sandor@aok.pte.hu (Z.S.); eva.szoke@aok.pte.hu (É.S.); 2 National Laboratory for Drug Research and Development (PharmaLab), 1117 Budapest, Hungary; 3 Institute of Pharmaceutical Chemistry, Faculty of Pharmacy, University of Pécs, 7624 Pécs, Hungary; 4 Chronic Pain Research Group (HUN-REN PTE), Hungarian Research Network, 7624 Pécs, Hungary; 5 Department of Pathology, Medical School, University of Pécs, 7624 Pécs, Hungary; semjen.david@pte.hu (D.S.); kajtar.bela@pte.hu (B.K.); 6 Department of Pharmacology, Faculty of Pharmacy, University of Pécs, 7624 Pécs, Hungary; 7 PharmInVivo Ltd., 7629 Pécs, Hungary

**Keywords:** prostate adenocarcinoma, Transient Receptor Potential Ankyrin 1, Transient Receptor Potential Vanilloid 1, viability, calcium influx

## Abstract

Transient receptor potential ankyrin 1 (TRPA1) and vanilloid 1 (TRPV1) have been implicated in cancer biology, but their functional relevance in prostate adenocarcinoma (PAC) remains unclear. Therefore, we characterized TRPA1/TRPV1 expression and function in benign prostatic hyperplasia (BPH), PAC tissues, and PAC cell lines with different characteristics and examined the effects of their agonists on cancer-cell viability. *TRPA1* and *TRPV1* transcripts were assessed by RNAscope in situ hybridization and RT-qPCR, channel activity by Fura-2-based Ca^2+^ imaging, and viability/proliferation by ATP-based luminescence and automated cell counting. *TRPA1* and/or *TRPV1* mRNAs were detected in PAC tissues and cell lines, but selective stimulation with allyl-isothiocyanate (AITC) or capsaicin did not evoke Ca^2+^ influx, indicating no detectable functional channel activity under our experimental conditions. Non-selective TRPA1 activators induced Ca^2+^ influx responses that were not inhibited by the receptor antagonist A-967079. AITC concentration dependently reduced viability and proliferation of the androgen-sensitive 22Rv1 and LNCaP cells, and these effects were not prevented by TRPA1 antagonists. Capsaicin had no effect at TRPV1-selective concentrations. Thus, transcript expression does not predict functional TRPA1/TRPV1 activity in PAC cells, and AITC reduces PAC-cell viability through TRPA1-independent mechanisms.

## 1. Introduction

Prostate cancer is one of the most prevalent malignancies worldwide and represents the second most frequently diagnosed cancer in men after lung cancer. Although most patients are diagnosed with localized disease, a substantial proportion eventually develop locally advanced, metastatic or castration resistant (stages III and IV) prostate adenocarcinoma (PAC), associated with poor clinical outcomes [1]. Since disease progression and therapeutic resistance remain major clinical challenges, there is a pressing need to identify novel molecular targets and develop more effective pharmacological strategies for the treatment of PAC [2].

The non-selective cation channels Transient Receptor Potential Ankyrin 1 (TRPA1) and Vanilloid 1 (TRPV1) are polymodal sensors that can be activated by a broad spectrum of chemically diverse irritants. TRPA1 is activated by allyl-isothiocyanate (AITC), thymol, hydrogen–sulfide, chlorine and aldehydes including formaldehyde, crotonaldehyde and cinnamaldehyde [3,4]. In contrast, TRPV1 is activated by capsaicin, protones, anadamide and lipoxygenase-derived metabolites [5]. Activation of these ion channels triggers Ca^2+^ influx and has been shown to modulate multiple cellular processes, such as gene expression, the secretion of bioactive mediators, cell proliferation and cell survival [6,7].

Although both ion channels are best known for their roles in nociception and neurogenic inflammation, accumulating evidence indicates that they also contribute to cancer development and progression [8,9,10,11]. In particular, TRPA1 has emerged as a potential regulator of tumor biology, with altered expression and functional activity reported in several malignancies [12].

TRPA1 mRNA and protein expression have been reported in normal human prostate tissue, with immunohistochemical staining observed in glandular epithelial cells [13]. However, the limited availability and insufficient validation of TRPA1 antibodies have raised concerns regarding the reliability of protein expression studies, making the precise localization and abundance of TRPA1 and TRPV1 in the prostate epithelium difficult to establish [14]. In PAC, functional TRPA1 protein expression has been detected in primary prostate stromal cells, while mRNA expression has been reported in several prostate cancer cell lines, including DU145 and PC-3 [15].

TRPV1 expression has also been reported in prostate tissues and cells, including benign prostatic hyperplasia (BPH), PAC tissues, and both androgen-sensitive and androgen-independent PAC cell lines [16,17,18]. Functional studies have demonstrated capsaicin-induced intracellular Ca^2+^-influx, which is inhibited by the TRPV1 antagonist capsazepine, suggesting the presence of functional TRPV1 channels [18]. Although capsaicin-induced alterations in cell viability have been interpreted as evidence of functional TRPV1 activity, most studies used exceptionally high capsaicin concentrations, raising the possibility that the observed cytotoxic effects were at least partially independent of TRPV1 activation [18,19,20].

Despite growing interest in the role of TRPA1 and TRPV1 in PAC, their expression patterns, functional activity, and biological significance remain incompletely understood. Therefore, we aimed to comprehensively characterize the TRPA1 and TRPV1 in human BPH and PAC tissues, as well as in PAC cell lines, in order to clarify their contribution to PAC and evaluate their therapeutic potential.

## 2. Materials and Methods

### 2.1. Cell Lines

The androgen receptor (AR)-negative prostate cancer cell lines DU145 (HTB-81™, RRID: CVCL_0105) and PC-3 (CRL-1435™, RRID: CVCL_0035), as well as the AR-positive prostate cancer cell lines LNCaP (CRL-1740™, RRID: CVCL_1379) and 22Rv1 (CRL-2505™, RRID: CVCL_1045), were used in this study. All cell lines were obtained from the American Type Culture Collection (ATCC; Manassas, VA, USA).

Cell cultures were routinely tested for mycoplasma contamination using a PCR-based assay and regularly monitored for microbial contamination. All cell lines remained free of contamination throughout the study.

Cells were cultured in RPMI 1640 medium (Lonza, Basel, Switzerland) supplemented with 10% heat-inactivated fetal bovine serum (Sigma-Aldrich, Burlington, MA, USA), 1% penicillin–streptomycin (Thermo Fisher Scientific, Waltham, MA, USA), 1% non-essential amino acids (Sigma-Aldrich, Burlington, MA, USA), and 2 mM L-glutamine (Gibco, Thermo Fisher Scientific, Waltham, MA, USA). Cell seeding densities were adjusted according to the requirements of each experimental assay and are specified in the corresponding sections below.

### 2.2. RNA Isolation and Real-Time Quantitative PCR Analysis

For gene expression analysis, 1 × 10^6^ cells were considered one biological sample. For each cell line, 6–7 independent wells were collected for isolation (*n* = 6–7). Cells were homogenized in 1 mL of TRI reagent (Molecular Research Center, Inc., Cincinnati, OH, USA), and the total RNA was isolated using the Direct-zol RNA MicroPrep Kit (Zymo Research, Irvine, CA, USA), according to the manufacturer’s instructions. To eliminate genomic DNA contamination, RNA samples were treated with 1 U DNase I. RNA concentration and purity were determined using a Genova Nano microvolume spectrophotometer (Jenway, Fisher Scientific, Loughborough, UK). Complementary DNA (cDNA) was synthesized from 500 ng of total RNA using the High-Capacity cDNA Reverse Transcription Kit (Applied Biosystems, Thermo Fisher Scientific, Waltham, MA, USA).

Gene expression analysis was carried out using FAM-labeled human *TRPA1* (assay number: Hs00175798_m1) and *TRPV1*-specific (assay number: Hs00218912_m1) TaqMan Gene Expression Assays (Thermo Fisher Scientific, Waltham, MA, USA) on a QuantStudio™ 5 Real-Time PCR System (Applied Biosystems, Thermo Fisher Scientific). Reactions were carried out in 96-well optical plates in three technical replicates using 1× SensiFAST™ Probe Lo-ROX Master Mix (Meridian Bioscience, Memphis, TN, USA), 400 nM TaqMan primer–probe assay, and 20 ng of cDNA per reaction. The geometric mean of the expression levels of human hypoxanthine phosphoribosyltransferase 1 (*HPRT1*) and importin 8 (*IPO8*) was used for normalization, as previously described, and the relative gene expression levels were calculated using the 2^−ΔΔCt^ method [21,22].

### 2.3. Transient Transfection of TRPA1 and TRPV1

Chinese hamster ovary (CHO) cells (RRID: CVCL_0213) were transiently transfected using TurboFect Transfection Reagent (Thermo Fisher Scientific, Cat. No. R0532). For heterologous expression of human TRPA1 and TRPV1, cells were transfected with pT31-hTRPA1 and pT33-hTRPV1 plasmids, respectively, in which gene expression is driven by a CMV promoter. For the transfection in 96-well μ-plates, transfection complexes were prepared by mixing 0.5 μg plasmid DNA with 1 μL TurboFect reagent in 25 μL Opti-MEM Reduced Serum Medium (Thermo Fisher Scientific, Waltham, MA, USA) per well. The DNA–TurboFect mixture was incubated at room temperature for 15 min before being added to the CHO cell suspension.

As a transient transfection protocol was employed, all functional experiments were conducted 24 h after transfection, as the expression of the transfected ion channels declined markedly after 48 h, rendering the cells unsuitable for subsequent experiments. The sample size was *n* = 6 per group, with each sample measured in duplicate across three independent experimental series.

### 2.4. Intracellular Ca^2+^ Influx Measurements

The functional activity of TRPA1 and TRPV1 was assessed in 22Rv1, LNCaP, PC-3, and DU145 cells by measuring agonist-induced changes in intracellular Ca^2+^ concentration using a CLARIOstar Plus microplate reader (BMG LABTECH, Ortenberg, Germany). Cells were seeded into 96 well ibiTreat plates (ibidi GmbH, Gräfelfing, Germany) at a density of 6 × 10^4^ cells/well. Wild-type CHO cells served as negative controls, whereas CHO cells stably expressing human TRPA1 and TRPV1 were used as positive controls. HEK293T cells (CRL-1573™, RRID:CVCL_0045) and HEK293T cells stably overexpressing human TRPM8 (hTRPM8), used as controls for TRPM8 functional assays, were kindly provided by Prof. Thomas Voets (KU Leuven, Leuven, Belgium). Vehicle-treated cells receiving the corresponding extracellular solution (NaCl, 160 mM; KCl, 2.5 mM; CaCl_2_, 1 mM; MgCl_2_, 2 mM; HEPES, 10 mM; glucose, 10 mM; pH 7.3) served as negative technical controls.

Cells were loaded with 2.5 μM Ca^2+^-sensitive fluorescent dye Fura-2 acetoxymethyl ester (Fura-2 AM, Invitrogen, Thermo Fisher Scientific, USA) for 45 min at 37 °C. Cells were then washed and incubated with the selective TRPV1 agonist capsaicin (0.1 µM, 0.3 µM, or 1 μM, Sigma-Aldrich, St. Louis, MO, USA), the selective TRPA1 agonist AITC (100 μM, Merck, Darmstadt, Germany), the non-selective TRPA1 agonists formalin (333 μM, 1000 µM, 3333 µM, or 10,000 µM, MOLAR Chemicals Kft., Halásztelek, Hungary), cinnamaldehyde (300 or 500 μM, Sigma-Aldrich, St. Louis, MO, USA), thymol (100 or 200 μM, Magilab Kft., Budapest, Hungary), or the non-selective TRPM8 agonist icilin (0.1 µM, 1 µM, 3 µM, Sigma-Aldrich, St. Louis, MO, USA). Fluorescence was monitored for 30 min following compound administration using alternating excitation wavelengths of 340 and 380 nm, with the emission collected at 510 nm. The specificity of TRPA1-mediated Ca^2+^ responses was verified using the selective TRPA1 antagonist A-967079 (Tocris Bioscience, Bristol, UK) which was applied before agonist stimulation and remained present throughout the experiment. Each treatment was tested in separate wells, with each well representing one independent experimental measurement. No wells were reused during the experiments. The sample size was *n* = 6 per group, with each sample measured in duplicate across three independent experimental series.

### 2.5. Androgen-Receptor Immunohistochemistry

The collection and use of human tissue samples were approved by the Regional Research Ethics Committee of the University of Pécs on 11 June 2026 (approval No. 92-PTERKEB2026/1). All procedures involving human samples were conducted in accordance with the approved protocol. PAC and BPH tissue specimens were obtained during routine surgical procedures for pathological diagnosis. Following completion of the diagnostic evaluation, the remaining surplus FFPE tissue samples from histopathologically diagnosed cases of PAC and BPH were provided by the Department of Pathology, Medical School, University of Pécs. To verify the histological localization of PAC and BPH tissue sections, standard hematoxylin and eosin (H&E) staining was performed on each slide.

AR-specific immunohistochemistry was performed using the Leica BOND-MAX automated staining system. Here, 5 μm thick sections were deparaffinized followed by heat-induced antigen retrieval. Endogenous peroxidase activity was blocked with H_2_O_2_ prior to incubation with mouse monoclonal anti-androgen receptor primary antibody (1:50, clone AR27, Leica Biosystems, AR-318-L-CE), followed by the Leica Post Primary reagent and Polymer detection reagent. Immunoreactivity was detected using 3,3′-diaminobenzidine (DAB) chromogen. Sections were subsequently counterstained with hematoxylin, coverslipped, and evaluated by light microscopy.

### 2.6. TRPA1 and TRPV1 RNAscope In Situ Hybridization

To detect TRPA1 and TRPV1-specific transcripts, the RNAscope Multiplex Fluorescent Reagent Kit v2 (ACD, Hayward, CA, USA) was used according to the manufacturer’s protocol. Cells were harvested, washed and resuspended in phosphate-buffered saline (PBS). Cell suspensions were loaded into cytofunnel assemblies mounted on SuperFrost™ Plus slides (Erie Scientific, Portsmouth, NH, USA) and centrifuged using a Shandon Cytospin 3 cytocentrifuge (Thermo Shandon, Runcorn, UK) at 500 rpm for 5 min. Following cytocentrifugation, the slides were air-dried in a horizontal position, fixed in 10% Neutral Buffered Formalin Fixative (30 min, Leica Biosystems, Wetzlar, Germany), and dehydrated.

Rehydration was carried out on both FFPE tissue sections (5 μm) cytospin slides, followed by H_2_O_2_ Protease Plus treatments. The slides containing the different cell lines were rehydrated and treated with Protease III.

Samples were hybridized with probes specific to human *TRPA1* (ACD, Cat. No. 311951), *TRPV1* (ACD, Cat. No. 415381), together with human 3-plex positive control probes (ACD, Cat. No. 320861) and 3-plex negative control probes (ACD, Cat. No. 320871). Signal amplification and fluorescent channel development were performed. Nuclei were counterstained with DAPI, and slides were mounted using ProLong Antifade Mountant.

Fluorescent images were acquired using a Nikon Eclipse Ti2-E confocal microscope (Auro-Science Consulting Kft., Budapest, Hungary) with a 60× oil-immersion objective. DAPI, Cy3 and Cy5 fluorophores were used to visualize nuclei, *TRPA1* and *TRPV1*. DAPI was excited at 405 nm, Cy3 at 550 nm, and Cy5 at 650 nm laser line. Blue (DAPI), green (*TRPA1*) and red (*TRPV1*) pseudocolors were assigned to the signals. Brightness/contrast were processed using (Fiji, 1.53c, NIH, Bethesda, MD, USA).

### 2.7. Cell Proliferation Assay by Cell Counting

The AR-positive PAC cell lines LNCaP and 22Rv1 cells were seeded into 24-well plates at a cell density of 30,000/well and treated with 1, 10, and 50 μM AITC, 100 nM, 1 μM, 10 μM, and 100 μM capsaicin, and the corresponding vehicle controls (0.01% or 0.05% DMSO) for 48 h. Following treatment, cells were detached using trypsin–EDTA solution (Lonza, Basel, Switzerland), and viable cells were quantified by trypan blue using a LUNA-II Automated Cell Counter (Logos Biosystems, Anyang-si, Gyeonggi-do, Republic of Korea).

### 2.8. Cell Viability Assays

22Rv1 and LNCaP were incubated with the specific TRPA1 activator AITC at concentrations of 1, 2, 10, 50, 100, or 200 μM, with the non-selective TRPA1 agonist thymol at concentrations of 100, 200, 400, 600, or 800 μM, and with the TRPV1 agonist capsaicin at concentrations of 0.1, 0.3, 1, 5, 10, 50, 100 or 200 μM for 48 h.

To assess whether the effect of the two agonists was specifically mediated by TRPA1 or TRPV1, cells were pretreated with the TRPA1 antagonists HC-030031 (50 µM) and A-967079 (1 µM) or the TRPV1 antagonist SB-366791 (1 µM) for 10 min prior to the addition of the agonist.

Briefly, cells were seeded into 96-well plates at a density of 5000 cells/well and cultured for 48 h following treatment. Cell viability was assessed using the CellTiter-Glo^®^ Luminescent Cell Viability Assay (Promega Corp., Madison, WI, USA), according to the manufacturer’s instructions. ATP-based luminescence intensity was measured using an EnSpire^®^ Multimode Plate Reader (PerkinElmer, Waltham, MA, USA). The sample size was *n* = 6–24 per group, based on 2–8 independent experimental series.

### 2.9. Statistical Analysis

Statistical analyses were performed using GraphPad Prism™ version 8 (GraphPad Software, San Diego, CA, USA). Basal mRNA expression levels among untreated cell lines and the intracellular Ca^2+^-influx data of specific time points of PAC cell lines (AITC, 5 min; formalin, 30 min; capsaicin, 10 min; thymol, 7 min; cinnamaldehyde, 30 min; icilin, 5 min) were analyzed using one-way ANOVA followed by Tukey’s multiple comparisons test. *p* < 0.05 was considered statistically significant in all cases. All sample size and *p* values are demonstrated in Tables S1–S13.

## 3. Results

### 3.1. TRPV1 mRNA Is Expressed in All PAC Cell Lines, Whereas TRPA1 mRNA Is Restricted to Androgen-Sensitive Cells

**Figure 1:**
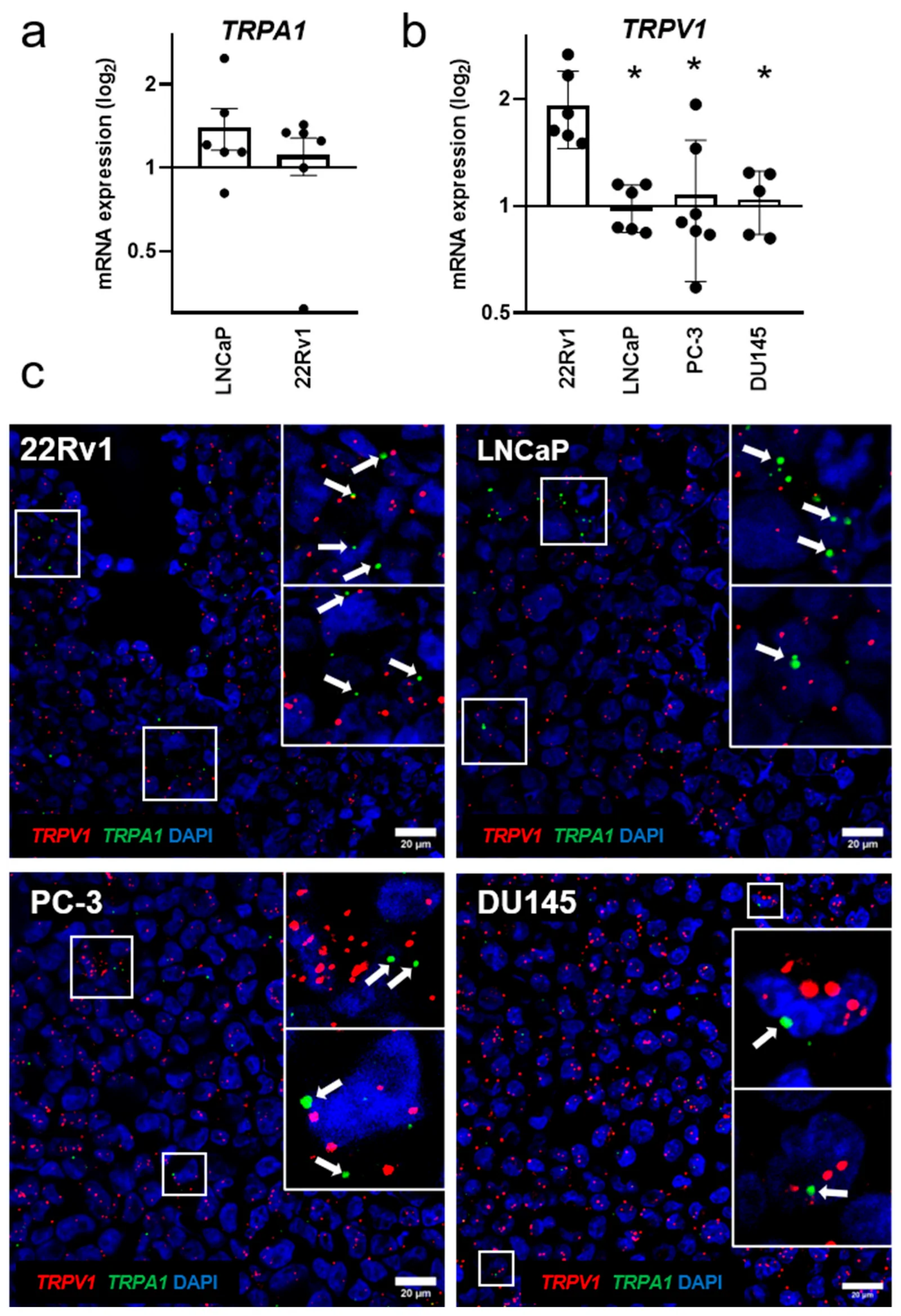
Expression of Transient Receptor Potential Ankyrin 1 (TRPA1) and Vanilloid 1 (TRPV1) mRNA in prostate adenocarcinoma cell lines. (**a**) TRPA1 mRNA was detected in the androgen-sensitive LNCaP and 22Rv1 cell lines, (**b**) whereas TRPV1 mRNA showed stable expression in all four cell lines. The mRNA expressions relative to the geometric mean of hypoxanthine phosphoribosyltransferase 1 and importin 8 were determined by TaqMan RT-qPCR in 22Rv1, LNCaP, PC-3, and DU145 cells. (**c**) Representative RNAscope in situ hybridization images showing TRPV1 (red dots) and TRPA1 (green dots, indicated by white arrows) mRNA transcripts in the indicated prostate cancer cell lines. Nuclei were visualized with DAPI (blue). Scale bar: 20 μm. Magnifications: 600×. Statistical analysis: *n* = 5–7; one-way ANOVA followed by Dunn’s multiple comparisons test; * *p* < 0.01 versus the corresponding control group.

qPCR analysis showed stable *TRPV1* mRNA in all four PAC cell lines, including the androgen-resistant PC-3 and DU145 cells as well as the androgen-sensitive LNCaP and 22Rv1 cells. The highest *TRPV1* mRNA expression was measured in 22Rv1 cells. In contrast, *TRPA1* mRNA expression was detected exclusively in the androgen-sensitive cell lines (Figure 1a,b, Tables S1 and S2), although the highly sensitive RNAscope in situ hybridization detected a limited number of *TRPA1* transcripts (Figure 1c).

### 3.2. TRPV1 and TRPA1 mRNA Are Expressed in Benign Prostatic Hyperplasia and Prostate Adenocarcinoma Tissues

**Figure 2:**
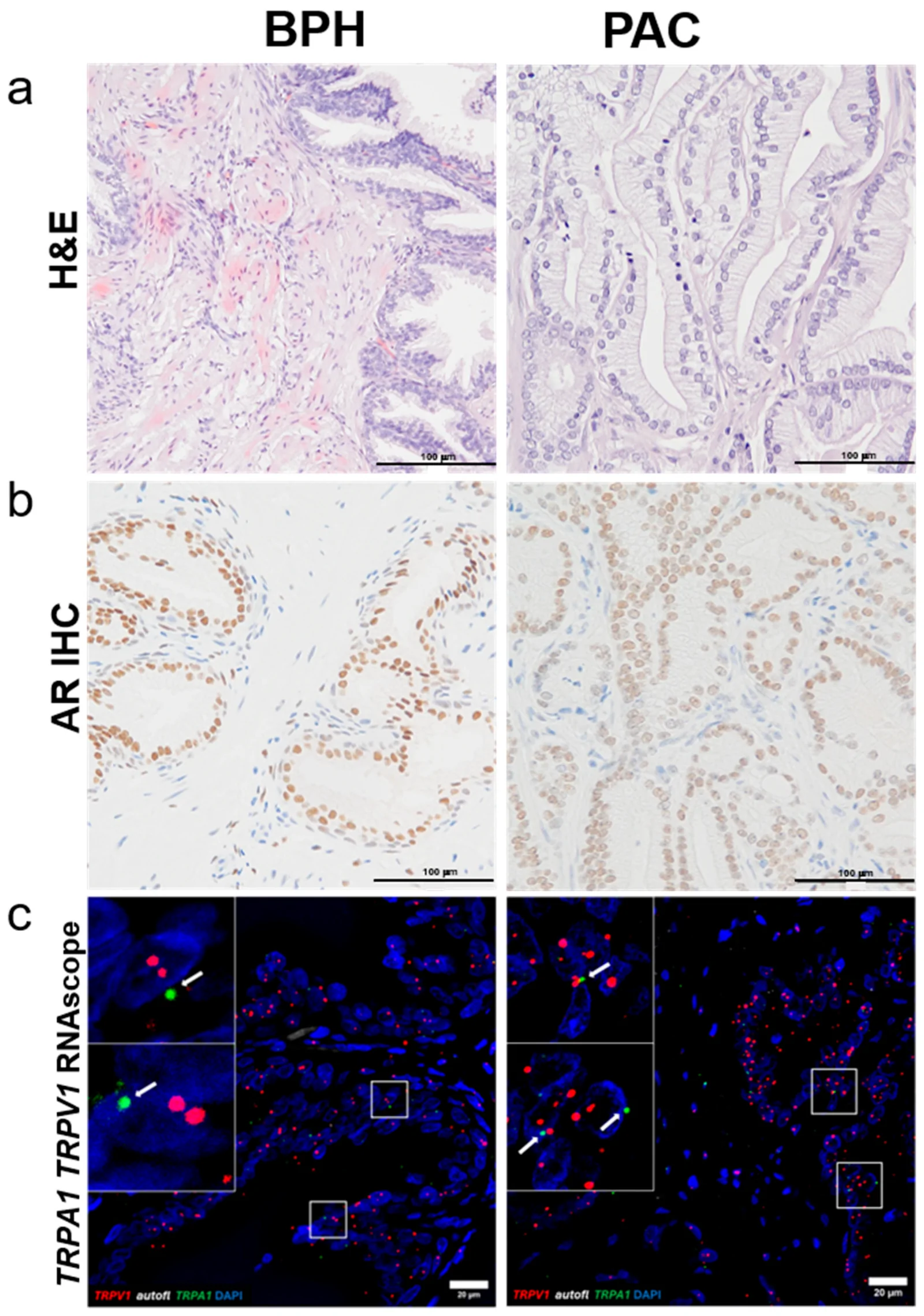
(**a**) Histopathological images, (**b**) androgen-receptor (AR) specific immunohistochemical analysis and (**c**) Transient Receptor Potential Vanilloid 1 (TRPV1) and Ankyrin 1 (TRPA1)-specific RNAscope in situ hybridization of benign prostatic hyperplasia (BPH) and prostate adenocarcinoma (PAC) tissues. AR-specific immunohistochemistry was performed together with hematoxylin and eosin (H&E) staining to reveal the tissue structure. *TRPV1* and *TRPA1* mRNA are expressed in BPH and PAC tissue samples detected by RNAscope in situ hybridization. Red dots represent *TRPV1*, while green dots and white arrows indicate *TRPA1* mRNA on panel (**c**), where nuclei were visualized with DAPI (blue). Scale bars: (**a**,**b**), 100 µM; (**c**), 20 µM. Magnifications: (**a**,**b**) 200×; (**c**) 600×.

Hematoxylin and eosin stained sections showed characteristic papillated glands composed of multiple cell layers within hyperplastic prostatic tissue. In contrast, PAC shows crowded microglandular glands lined by a single layer of epithelial cells with occasional prominent nucleoli (Figure 2a). Androgen receptor immunohistochemistry, with hematoxylin counterstaining, demonstrated diffuse nuclear positivity in both hyperplastic prostatic glands and PAC (Figure 2b).

*TRPV1* and *TRPA1* mRNA are present in the epithelial cells of androgen receptor-positive BPH and PAC tissue samples as detected with RNAscope in situ hybridization. *TRPA1* was present with relatively low copy numbers in both tissue types, while *TRPV1* mRNA exhibited high expression levels (Figure 2c).

### 3.3. Formalin, but Not AITC, Increases Intracellular Ca^2+^ Levels Independently of TRPA1

**Figure 3:**
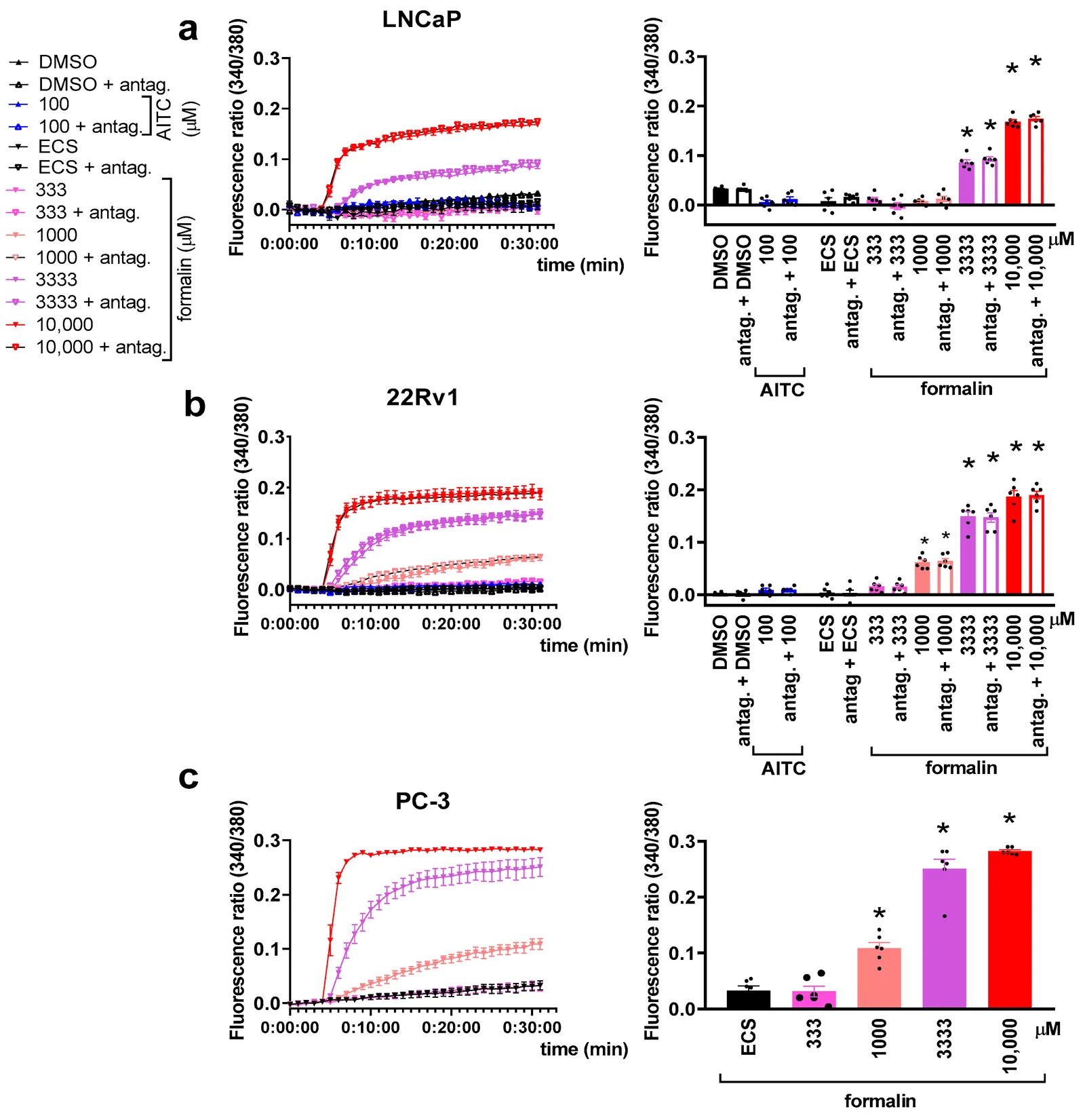
Effects of Transient Receptor Potential Ankyrin 1 (TRPA1) agonists on intracellular Ca^2+^ levels in prostate cancer cell lines. Changes in intracellular Ca^2+^ concentrations were recorded in (**a**) LNCaP, (**b**) 22Rv1, and (**c**) PC-3 cells following stimulation with the selective TRPA1 agonist allyl isothiocyanate (AITC) or the non-selective TRPA1 agonist formalin with or without the presence of 1 µM selective TRPA1 antagonist A-967079 (antag.) for 30 min. Data are presented as means ± SEM (*n* = 6, from 3 independent experimental series). Statistical analysis: one-way ANOVA followed by Tukey’s multiple comparisons test; * *p* < 0.001 versus the corresponding control group.

Treatment with 100 μM AITC did not alter the intracellular Ca^2+^ levels in either LNCaP or 22Rv1 cell lines. In contrast, formalin induced a concentration-dependent increase in intracellular Ca^2+^ concentration across the entire concentration range tested. A significant Ca^2+^-influx was measured following treatment with 3333 µM and 1000 µM formalin in 22Rv1 and LNCaP cells, respectively. However, this response was not inhibited by the selective TRPA1 antagonist A-967079, indicating that the detected Ca^2+^ signal was independent of TRPA1 activation (Figure 3a,b, Table S3).

To prove the TRPA1-independent nature of the formalin-evoked intracellular Ca^2+^ response, measurements were conducted using TRPA1-negative PC-3 cells in which a significant Ca^2+^ signal was detected at 1000 µM (Figure 3c, Table S3). To validate the assay, CHO cells stably expressing human TRPA1 were stimulated with 100 µM AITC, which induced a robust increase in the intracellular Ca^2+^ concentration (Figure S1a, Table S7).

### 3.4. Lack of Functional TRPV1 Responses in All PAC Cell Lines

**Figure 4:**
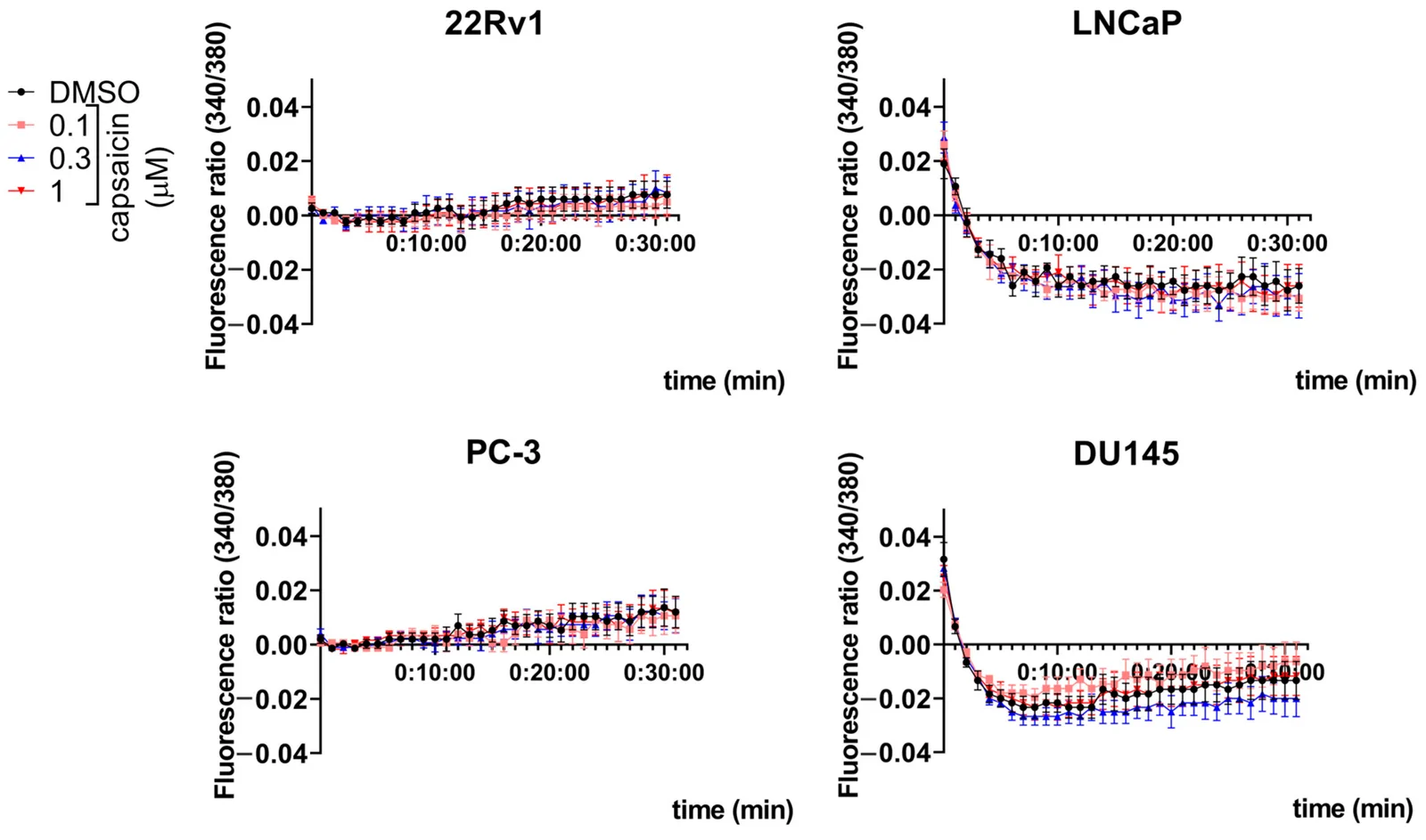
There was no effect of capsaicin on intracellular Ca^2+^ concentration in prostate adenocarcinoma cell lines. Intracellular Ca^2+^ levels were recorded for 30 min in 22Rv1, LNCaP, PC-3 and DU145 cells following stimulation with the selective TRPV1 agonist capsaicin. Data are presented as means ± SEM (*n* = 6, from 3 independent experimental series). Statistical analysis: one-way ANOVA followed by Tukey’s multiple comparisons test.

Despite stable TRPV1 mRNA expression, capsaicin failed to induce Ca^2+^-influx in any of the examined PAC cell lines (Figure 4, Table S4). To validate the assay, intracellular Ca^2+^ measurements were performed on human TRPV1-expressing transfected CHO cells. As expected, 1 μM capsaicin induced a robust Ca^2+^-influx in these cells (Figure S1b, Table S8).

### 3.5. Both Thymol and Cinnamaldehyde Induce a TRPA1-Independent Ca^2+^-Influx in PAC Cells

**Figure 5:**
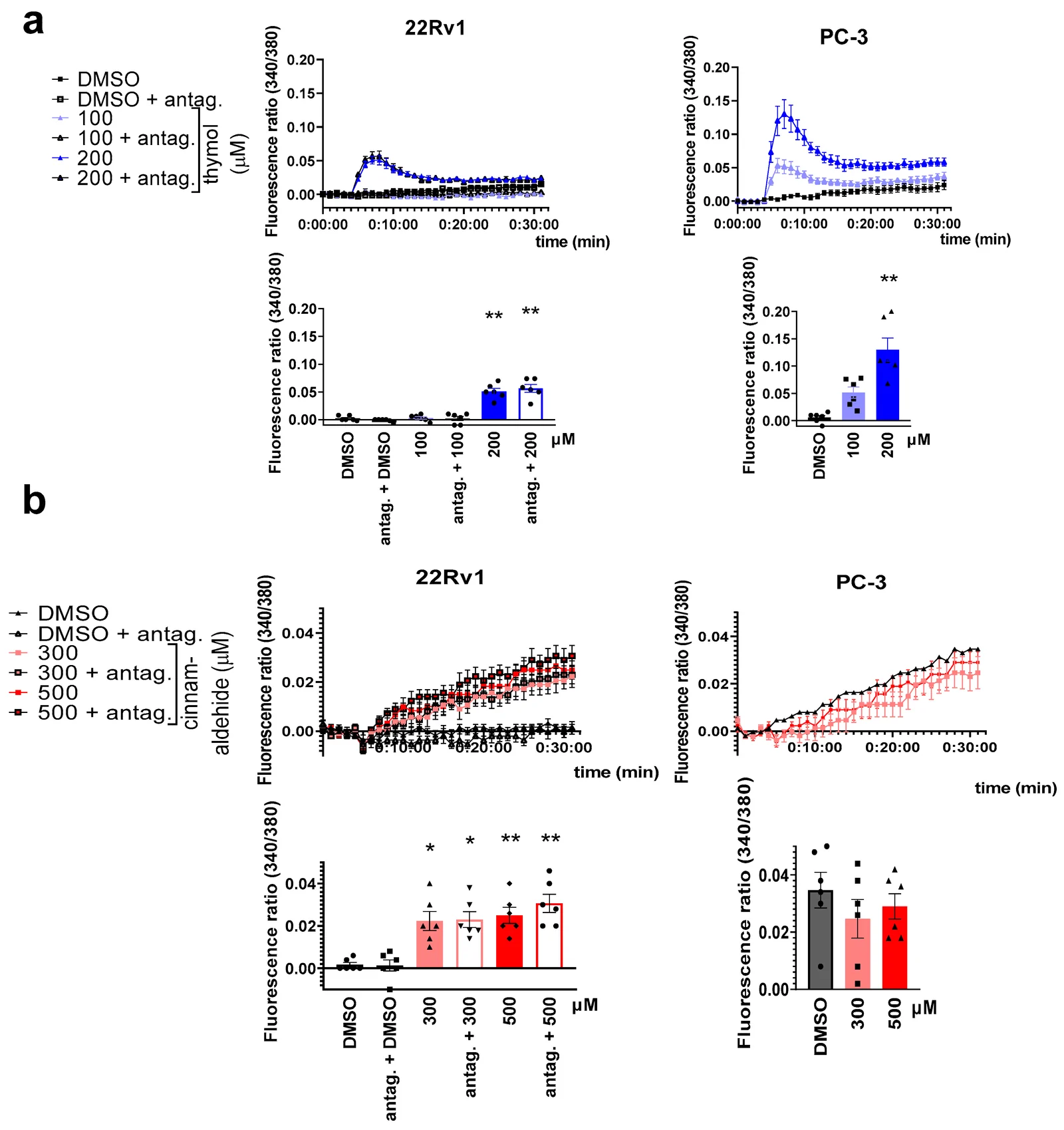
Effects of the non-selective TRPA1 agonists thymol and cinnamaldehyde on intracellular Ca^2+^ levels in prostate cancer cell lines. Changes in intracellular Ca^2+^ levels were measured in 22Rv1 and PC-3 cells following stimulation with (**a**) thymol or (**b**) cinnamaldehyde for 30 min with or without the presence of 1 µM selective TRPA1 antagonist A-967079 (antag.). Data are presented as means ± SEM (*n* = 6, from 3 series of independent experiments). Statistical analysis was performed using one-way ANOVA followed by Tukey’s multiple comparisons test. * *p* < 0.01, ** *p* < 0.001 versus the corresponding control group.

Treatment with 200 μM thymol and 300 μM cinnamaldehyde induced an increase in the intracellular Ca^2+^ concentration in 22Rv1 cells, which was not inhibited by the TRPA1 antagonist A-967079. This non-specific activation of thymol was also observed in the TRPA1-negative PC-3 cells, in which cinnamaldehyde did not induce Ca^2+^ accumulation (Figure 5a,b, Table S5).

To validate the Ca^2+^-influx responses induced by cinnamaldehyde and thymol, experiments were performed in both wild-type and TRPA1-transfected CHO cells. Here, 300 µM of cinnamaldehide increased the intracellular Ca^2+^ concentrations of TRPA1-transfected CHO cells, whereas 500 μM of cinnamaldehyde induced a decrease in the intracellular Ca^2+^ levels of CHO cells lacking functional TRPA1. In contrast, thymol induced Ca^2+^-influx in wild-type CHO cells only at 200 µM, whereas a robust increase in intracellular Ca^2+^ concentration was already observed at 100 µM in TRPA1-transfected CHO cells (Figure S1a, Table S7). To exclude the possibility of thymol-induced Ca^2+^-influx being mediated by TRPM8 activation, cells were stimulated with the selective TRPM8 agonist icilin; however, no intracellular Ca^2+^ response was detected (Figure S2, Table S6) TRPM8-expressing HEK cells exhibited a marked elevation in intracellular Ca^2+^ concentration following icilin stimulation compared to negative control HEK cells (Figure S1c, Table S9).

### 3.6. Allyl Isothiocyanate, Capsaicin and Thymol Reduce the Viability of LNCaP and 22Rv1 Cells in a Concentration-Dependent Manner

**Figure 6:**
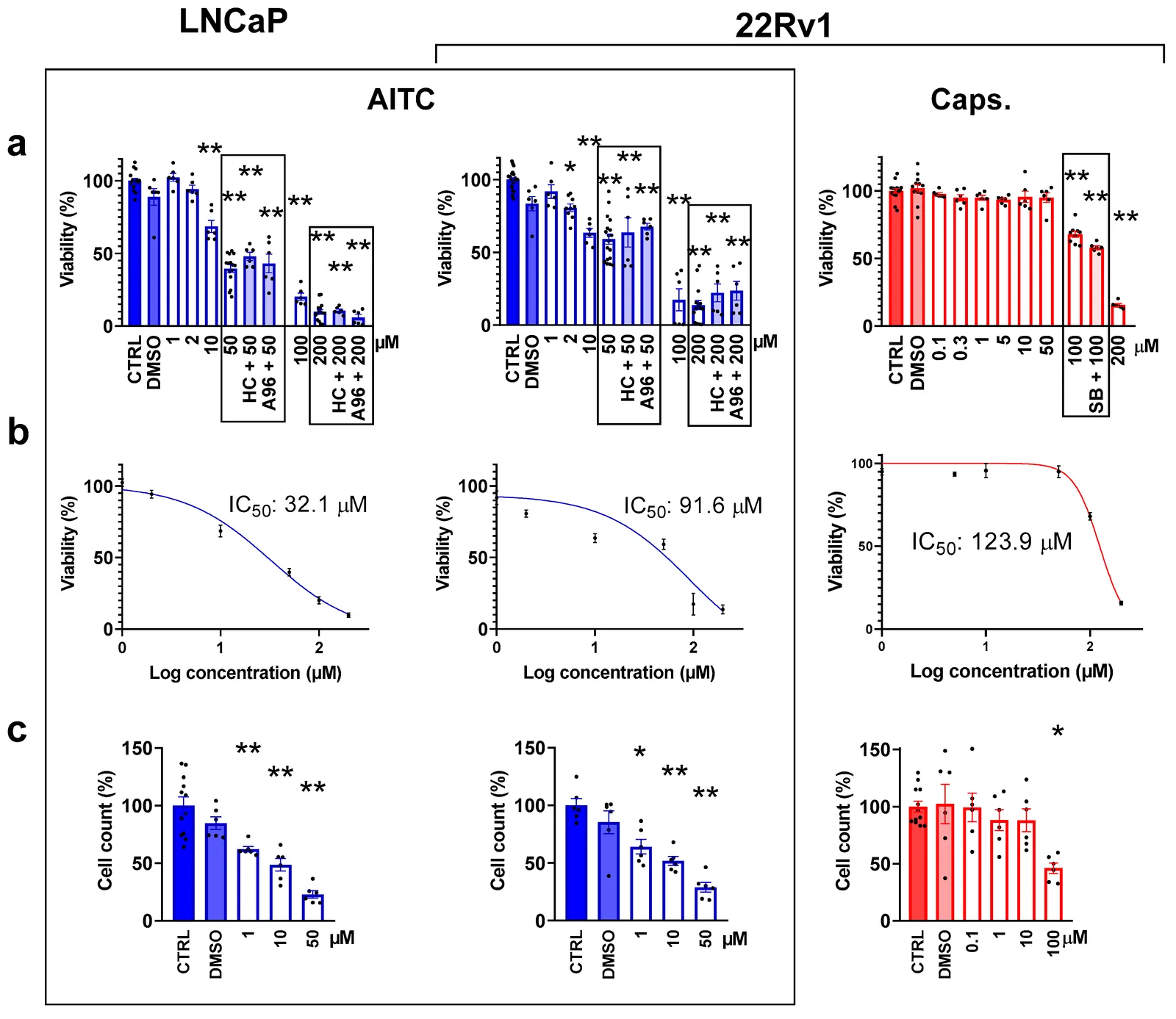
Viability decreasing effects of allyl isothiocyanate (AITC) and capsaicin on prostate adenocarcinoma cell lines. (**a**) ATP-based viability decreases in LNCaP and 22Rv1 cells following 48 h of incubation with capsaicin and AITC in the absence or presence of the TRPA1 antagonists HC-030031 (HC, 50 µM) or A-967079 (A96, 1 µM) or TRPV1 antagonist SB-366791 (1 µM). Boxes indicate agonist–antagonist co-treatment conditions. (**b**) Concentration–response curves showing the effect of AITC and capsaicin in LNCaP and 22Rv1 cells. (**c**) Direct cell counts of viable LNCaP and 22Rv1 cells following 48 h of treatment with AITC or capsaicin. Data are presented as means ± SEM (*n* = 6–24 per group, obtained from 2–8 independent experimental series). Statistical analysis: one-way ANOVA followed by Tukey’s multiple comparisons test. * *p* < 0.01 and ** *p* < 0.001 versus the corresponding control group.

Following 48 h of AITC treatment, the cell proliferation and viability decreased in both LNCaP and 22Rv1 cells in a concentration-dependent manner, reaching statistical significance at 10 µM and 2 µM, respectively (Tables S10 and S11). The IC_50_ values for AITC were 32.8 µM in LNCaP and 91.6 µM in 22Rv1 cells (Figure 6a,b). Co-treatment with the TRPA1 antagonists HC-030031 or A-967079 did not significantly attenuate the AITC-induced reduction in cell viability at either 50 or 200 µM AITC in either cell line (Table S10).

TRPV1-selective concentrations (0.1, 0.3, 1 and 10 µM) of capsaicin did not affect the proliferation of 22Rv1 cell line, reaching statistical significance at 100 µM (Figure 6c, Table S13). However, capsaicin significantly reduced the ATP levels in LNCaP and 22Rv1 cells at concentrations of 5 and 100 µM, respectively, and the effect was not attenuated by SB-366791, suggesting a TRPV1-independent mechanism (Figure S3, Table S12).

Thymol reduced 22Rv1 cell viability in a concentration-dependent manner, with a significant reduction observed at 400 µM, and the calculated IC_50_ value was 442.2 µM (Figure S4). Co-treatment with the TRPA1 antagonist A-967079 did not significantly attenuate this effect, suggesting a TRPA1-independent mechanism.

## 4. Discussion

This is the first study demonstrating stable TRPA1 and TRPV1 mRNA expression in BPH and PAC tissues, as well as in androgen-sensitive PAC cell lines, with substantially higher TRPV1 abundance than TRPA1. Furthermore, TRPV1 mRNA was also detected in the androgen-resistant PAC cell lines DU145 and PC3.

Reliable detection of TRPA1 and TRPV1 protein abundance for both immunohistochemistry and Western blotting still remains a huge technical problem due to the lack of selective antibodies [14,23,24]. Measurement of the Ca^2+^ influx in response to selective agonists is widely used to assess the activity of Ca^2+^-permeable channels, providing indirect evidence for functional channel expression [23,24]. These assays are therefore suitable for investigating whether ion channels mediate a functional response in cultured cell lines. Therefore, pharmacological characterization is essential to prove the presence of functional ion channel proteins. Despite detectable TRPA1 and TRPV1 transcripts measured by both qPCR and the sensitive RNAscope in situ hybridization, selective activation of either channel with AITC or capsaicin failed to induce intracellular Ca^2+^-influx in any of the examined PAC cell lines. In contrast, the non-selective activators formalin, thymol and cinnamaldehyde significantly induced a Ca^2+^ increase. However, these responses were not prevented by the selective TRPA1 antagonist A-967079, indicating that the observed signals were independent of TRPA1 activation.

Several mechanisms may explain the non-specific Ca^2+^ responses induced by these compounds. Due to its reactivity, formalin can covalently modify proteins and membrane lipids, leading to cellular injury and disruption of intracellular Ca^2+^ homeostasis [25]. In addition, formaldehyde has been reported to increase intracellular Ca^2+^ levels through the activation of NMDA receptors and T-type Ca^2+^ channels, as well as by inducing mitochondrial dysfunction and the production of reactive oxygen species (ROS) leading to mitochondrial overload [26,27,28,29].

Thymol has been shown to elevate the IC Ca^2+^ level not through TRP channels but through phospholipase-C -dependent Ca^2+^ release from the endoplasmic reticulum together with PKC-sensitive store-operated Ca^2+^ entry. Furthermore, the reduced cell viability was not affected by the intracellular Ca^2+^ chelator BAPTA/AM, indicating that cell death was not dependent from the increased intracellular Ca^2+^ levels [30]. Previous studies have demonstrated that thymol reduces LNCaP cell viability by activating caspase-3 and modulating apoptotic-related gene expression [31]. These data support our conclusion that the cytotoxic effect of thymol is independent of TRPA1. Although cinnamaldehyde is widely used as a selective TRPA1 agonist, in our experiment, it increased the intracellular Ca^2+^-influx through TRPA1-independent mechanisms, as demonstrated by the lack of inhibition by A-967079. Due to its lipophilicity, cinnamaldehyde may alter the membrane fluidity and permeability, affecting the activity of other ion channels, including voltage-gated Ca^2+^ channels [32].

The lack of TRPA1-mediated Ca^2+^ signaling despite detectable mRNA expression is supported by previous studies demonstrating that mRNA abundance correlates weakly to moderately with the protein level, depending on the tissue and cell type [33]. This discrepancy may result from post-transcriptional and post-translational regulation, low protein abundance, impaired trafficking to the plasma membrane, or expression of non-functional splice variants [6,34,35].

AITC significantly reduced both the viability and proliferation of 22Rv1 and LNCaP cells; however, neither HC-030031 nor A-967079 prevented this effect, indicating that the cytotoxic action of AITC is largely TRPA1-independent.

AITC is a highly electrophilic isothiocyanate whose biological activity extends beyond TRPA1 agonism through the reversible covalent modification of proteins, disruption of cellular redox homeostasis, activation of stress-response pathways such as KEAP1–NRF2, MAPK signaling, and induction of mitochondrial dysfunction accompanied by ROS production, ultimately resulting in antiproliferative and pro-apoptotic effects, as shown in several cancer models [36,37,38,39].

In addition, AITC has been reported to impair cell adhesion, induce cytoskeletal remodeling and cell rounding, and promote cell detachment before apoptosis, consistent with the reduction in viable cell numbers observed in the present study [40].

In our hands, the TRPV1-selective concentrations of capsaicin did not alter either the intracellular Ca^2+^ concentration or the ATP-based viability or cell number of PAC cell lines, suggesting that TRPV1 is not involved in prostate cancer cell biology [17,18,41]. However, higher concentrations of capsaicin (50, 100 and 200 μM) reduced the viability of 22Rv1 and LNCaP PAC cells. These effects were not prevented by the TRPV1 antagonist SB-366791, further supporting the conclusion that capsaicin-induced cell viability reduction at higher concentrations occurs through TRPV1-independent mechanisms. This conclusion is consistent with a previous study, in which highly sensitive radioactive Ca^2+^-influx assay failed to detect functional TRPV1 in PAC cell lines in TRPV1-selective concentrations, concluding that the cytotoxic effects of capsaicin occur only at substantially higher concentrations [20]. In contrast, a previous study has demonstrated both TRPV1 mRNA and protein expression in BPH and PAC tissues, as well as in DU145, PC3 and LNCaP cell lines [42,43]. Capsaicin has also been shown to reduce proliferation in LNCaP and PC3 cells; however, these effects were not reversed by TRPV1 antagonists, and the concentrations exceeded the range considered selective for TRPV1 activation [44]. Together, these data suggest that these effects of capsaicin are mediated, at least in part, through TRPV1-independent signaling pathways [19].

*TRPV1* expression showed no significant difference between healthy prostate tissue obtained from transplantation donors and BPH, while increased expression was observed in prostate carcinoma, correlating with the tumor grade [42]. These findings suggest that TRPV1 expressional changes may be associated with prostate cancer progression. Although these data might suggest that TRPV1 could be an early molecular marker for transition to malignant states, further studies involving well-characterized and disease-free prostate tissue and premalignant lesions are required to clarify its potential prognostic value.

An important limitation of the present study is the lack of healthy prostate tissue for comparison. Although BPH represents a non-malignant condition, it cannot be considered as a healthy organ. A further limitation of the present study is the lack of a selective antibody for the reliable detection of TRPA1 and TRPV1 protein abundance as described by us and several other groups [14,45,46,47,48]. Consequently, our conclusions regarding these channels are based primarily on transcript-level expression and functional pharmacological assays.

Our findings emphasize the importance of complementing transcriptomic and pharmacological studies of TRPA1 and TRPV1 with functional assays, as this integrated approach enables the discrimination of ion channel-specific cellular processes from the broader biological actions. Furthermore, the present data suggest that AITC and its derivatives may represent promising agents for the treatment of PAC. However, their antitumor effects appear to be mediated at least in part through TRPA1-independent mechanisms, which require further investigation.

## Data Availability

The datasets used and/or analyzed during the current study available from the corresponding author on reasonable request.

## Supplementary Materials

The following supporting information can be downloaded at https://www.mdpi.com/article/10.3390/cells15211986/s1, Figure S1: Positive and negative control Ca^2+^ influx measurements in *TRPA1*- and *TRPV1*-transfected naive CHO cells and TRPM8 expressing and non-expressing HEK cells; Figure S2: No change in intracellular Ca^2+^ levels induced by TRPM8 agonist icilin in prostate cancer cell lines; Figure S3: Viability decreasing effect of capsaicin in the LNCaP prostate adenocarcinoma cell line; Figure S4: Viability decreasing effect of thymol in the 22Rv1 prostate adenocarcinoma cell line; Table S1: Statistical analysis of TRPV1 expression of prostate adenocarcinoma cell lines; Table S2: Statistical analysis of TRPA1 expression of prostate adenocarcinoma cell lines; Table S3: Statistical analysis of the effects of allyl-isothiocyanate (AITC) and formalin on intracellular Ca^2+^ levels in prostate cancer cell lines; Table S4: Statistical analysis of the effect of capsaicin on intracellular Ca^2+^ levels in prostate cancer cell lines; Table S5: Statistical analysis of the effect of cinnamaldehyde and thymol on intracellular Ca^2+^ levels in prostate cancer cell lines; Table S6: Statistical analysis of the effect of icilin on intracellular Ca^2+^ levels in prostate cancer cell lines; Table S7: Statistical analysis of the effect of AITC, formalin, thymol and cinnamaldehyde on intracellular Ca^2+^ levels in TRPA1-transfected and naive CHO cells; Table S8: Statistical analysis of the effect of capsaicin on intracellular Ca^2+^ levels in TRPV1-transfected and naive CHO cells; Table S9: Statistical analysis of the effect of icilin on intracellular Ca^2+^ levels in TRPM8-expressing and naïve HEK cells; Table S10: Statistical analysis of the effect of AITC on ATP-based viability of prostate adenocarcinoma cell lines; Table S11: Statistical analysis of the effect of AITC on cell counts of prostate adenocarcinoma cell lines; Table S12: Statistical analysis of the effect of capsaicin on ATP-based viability of prostate adenocarcinoma cell lines; Table S13: Statistical analysis of the effect of capsaicin on the cell counts of 22Rv1 prostate adenocarcinoma cell line.

## Abbreviations

The following abbreviations are used in this manuscript:

AITC	allyl isothiocyanate
AR	androgen receptor
BPH	benign prostatic hyperplasia
CHO	Chinese hamster ovary
cDNA	complementary DNA
DAB	diaminobenzidine
H&E	hematoxylin and eosin
PAC	prostate adenocarcinoma
PBS	phosphate buffered saline
TRPA1	Transient Receptor Potential Ankyrin 1
TRPV1	Transient Receptor Potential Vanilloid 1
