# Supplementary material for: Transient Receptor Potential Ankyrin 1 and Vanilloid 1 Receptor mRNAs Are Not Translated to Functional Ion Channels in Human Prostate Adenocarcinoma Cells
Source: Cells. 2026 Oct 30;15(21):1986. doi: 10.3390/cells15211986 (PMC13761908; doi:10.3390/cells15211986)
Supplement: Supplementary file 1 [file cells-15-01986-s001.zip › cells-4595408-supplementary.pdf]

Supplementary material Steib et al.

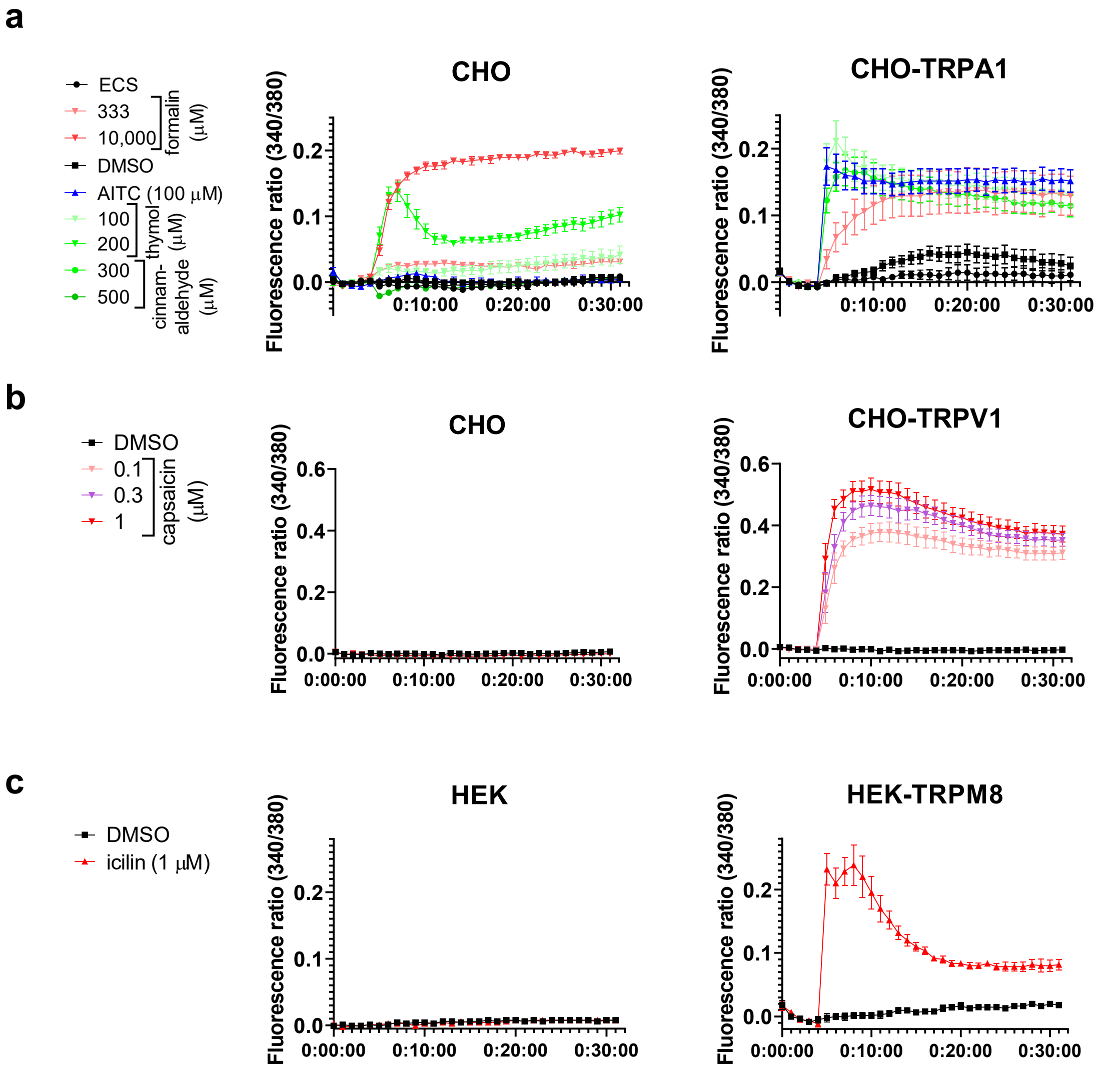

Figure S1. Positive and negative control Ca²⁺ influx measurements in *TRPA1*- and *TRPV1*-transfected, naive CHO cells, TRPM8 expressing and, non-expressing HEK cells. Ca^{2+}-influx induced by thymol, cinnamaldehyde, formaldehyde, AITC and capsaicin was evaluated in (a) TRPA1-transfected, (b) TRPV1-transfected, and (a, b) naive CHO cells. (c) Icilin induced Ca^{2+}-influx was measured in HEK and TRPM8 expressing HEK cells. Data are presented as means ± SEM (n = 6).

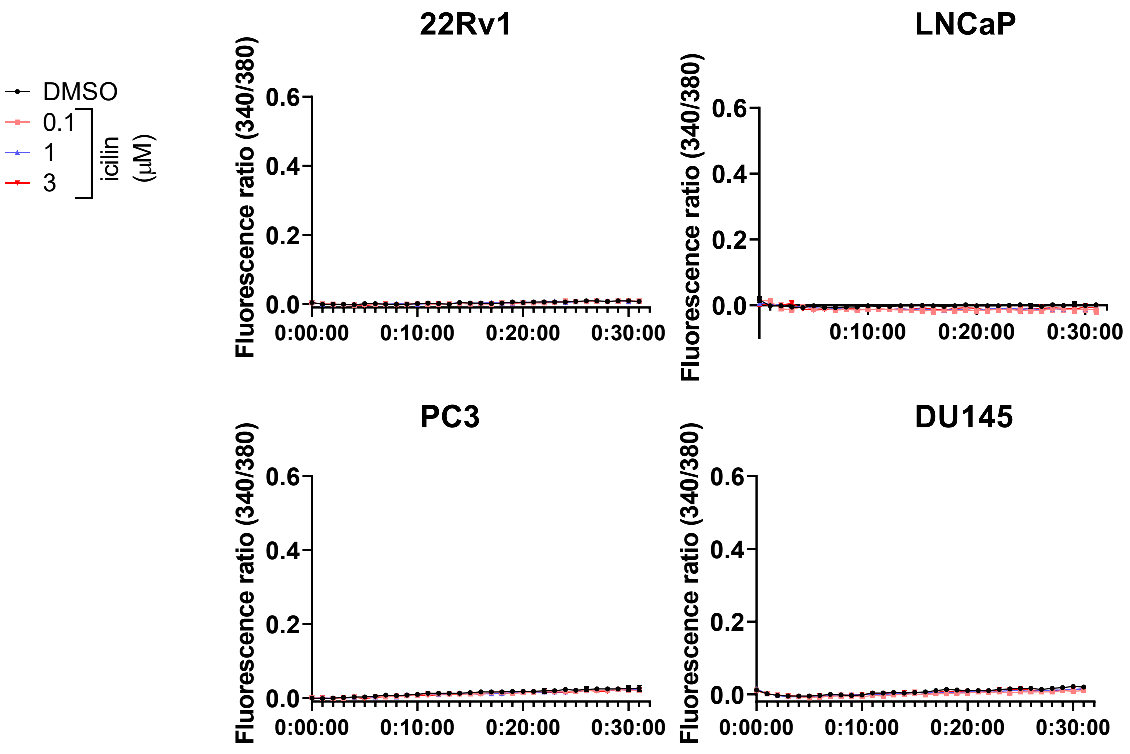

Figure S2. No change in intracellular Ca^{2+} levels induced by TRPM8 agonist icilin in prostate cancer cell lines. Stimulation with the selective TRPM8 agonist icilin for 30 min did not elicit detectable changes in intracellular Ca^{2+} levels in 22Rv1, LNCaP, PC-3, or DU145 cells. Data are presented as means ± SEM (n = 6). Statistical analysis was performed using one-way ANOVA followed by Tukey's multiple comparisons test.

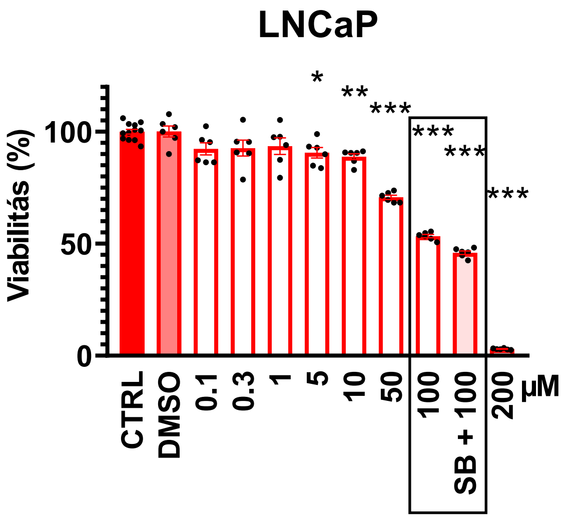

Figure S3. Viability decreasing effect of capsaicin in the LNCaP prostate adenocarcinoma cell line. ATP-based viability of LNCaP cells following 48 h incubation with capsaicin. Data are presented as means ± SEM (n = 6-12). Statistical analysis was performed using one-way ANOVA followed by Tukey's multiple-comparisons test. *$p < 0.05$, **$p < 0.01$ and ***$p < 0.001$ was considered as statistically significant..

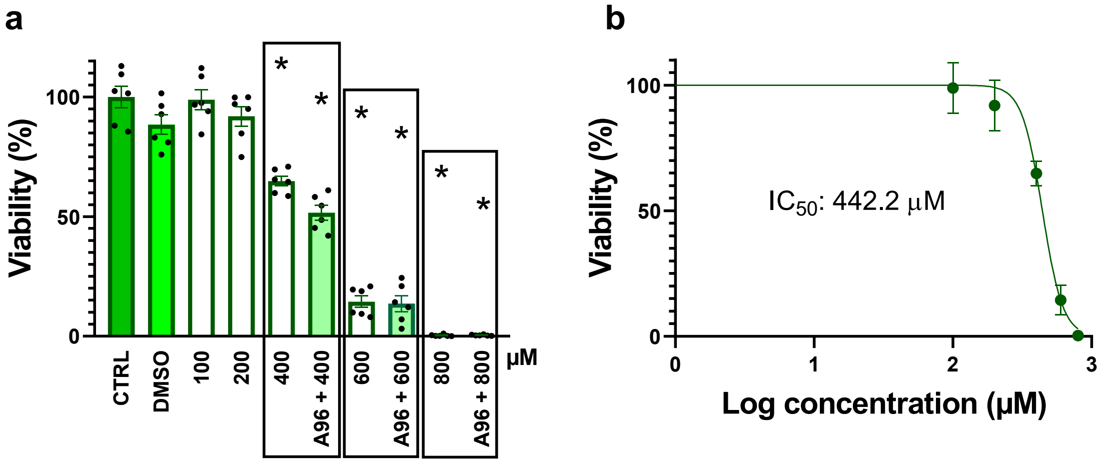

Figure S4. Viability decreasing effect of thymol in the 22Rv1 prostate adenocarcinoma cell line. (a) ATP-based viability decrease and **(b)** concentration-response curve of 22Rv1 cells following 48 h incubation with thymol. Data are presented as means ± SEM (n = 6). Statistical analysis: one-way ANOVA followed by Tukey's multiple comparisons test. *$p < 0.0001$ was considered as statistically significant.

Table S1. Statistical analysis of TRPV1 expression of prostate adenocarcinoma cell lines. The table shows the statistical data generated by one-way ANOVA followed by Tukey's multiple-comparisons test of n=5-7/group. *$p < 0.01$ was considered as statistically significant.

Cell line	22Rv1	LNCaP	PC-3	DU145
**22Rv1** n=6		**0.0015** *	**0.0024** *	**0.0038** *
**LNCaP** n=6			0.9802	0.9961
**PC-3** n=7				0.9990
**DU145** n=5				

Table S2. Statistical analysis of TRPA1 expression of prostate adenocarcinoma cell lines. The table shows the statistical data generated by one-way ANOVA followed by Tukey's multiple-comparisons test of n=6/group. $p < 0.05$ was considered as statistically significant.

Cell line	**LNCaP** n=6
**22Rv1** n=6	0.3579

Table S3. Statistical analysis of the effects of allyl-isothiocyanate (AITC) and formalin on intracellular Ca^{2+} levels in prostate cancer cell lines. The analysis was based on fluorescence ratios recorded 1 min and 25 min after the addition of AITC and formalin, respectively. The table shows the statistical data generated by one-way ANOVA followed by Tukey's multiple-comparisons test, n=6/group. *p < 0.001 was considered as statistically significant.

Cell line	100 µM AITC vs. DMSO		333 µM formalin vs. ECS		1,000 µM formalin vs. ECS		3,333 µM formalin vs. ECS		10,000 µM formalin vs. ECS	
	-A96	+A96	-A96	+A96	-A96	+A96	-A96	+A96	-A96	+A96
**22Rv1**	>0,9999	>0,9999	0,9768	0,9857	**<0,0001** *	**<0,0001** *	**<0,0001** *	**<0,0001** *	**<0,0001** *	**<0,0001** *
-A96 vs. +A96	>0,9999		>0,9999		>0,9999		>0,9999		>0,9999	
**LNCaP**	>0,9999	>0,9999	>0,9999	0,9804	0,9897	>0,9999	**<0,0001** *	**<0,0001** *	**<0,0001** *	**<0,0001** *
-A96 vs. +A96	0,9997		0,8529		>0,9999		0,9992		0,9995	
**PC-3**			>0,9999		0,0004		**<0,0001** *		**<0,0001** *	

Table S4. Statistical analysis of the effect of capsaicin on intracellular Ca^{2+} levels in prostate cancer cell lines. The analysis was based on fluorescence ratios recorded 5 min after the addition of capsaicin. The table shows the statistical data generated by one-way ANOVA followed by Tukey's multiple-comparisons test, n=6/group. *p < 0.001 was considered as statistically significant.

Cell line	0.1 µM capsaicin vs. DMSO	0.3 µM capsaicin vs. DMSO	1 µM capsaicin vs. DMSO
**22Rv1**	0,9660	0,9994	0,9912
**LNCaP**	>0,9999	>0,9999	0,8703
**PC-3**	0,9623	0,9882	0,9882
**DU145**	0,7794	0,8105	0,9907

Table S5. Statistical analysis of the effect of cinnamaldehyde and thymol on intracellular Ca^{2+} levels in prostate cancer cell lines. The analysis was based on fluorescence ratios recorded 25 min and 2 min after the addition of cinnamaldehyde and thymol respectively. The table shows the statistical data generated by one-way ANOVA followed by Tukey's multiple-comparisons test, n=6/group. *p < 0.001 was considered as statistically significant.

Cell line	300 µM cinnamaldehyde vs. DMSO		500 µM cinnamaldehyde vs. DMSO		100 µM thymol vs. DMSO		200 µM thymol vs. DMSO	
	-A96	+A96	-A96	+A96	-A96	+A96	-A96	+A96
**22Rv1**	**0,0032** *	**0,0022** *	**0,0007** **	**<0,0001** **	>0,9999	>0,9999	**<0,0001** **	**<0,0001** **

-A96 vs. +A96	>0,9999	0,8622	>0,9999	0,9276
**PC-3**	0,4707	0,7782	0,0851	**<0,0001 ****

Table S6. Statistical analysis of the effect of icilin on intracellular Ca^{2+} levels in prostate cancer cell lines. The analysis was based on fluorescence ratios recorded 1min after the addition of icilin. The table shows the statistical data generated by one-way ANOVA followed by Tukey's multiple-comparisons test, n=6/group. *p < 0.001 was considered as statistically significant.

Cell line	0.1 µM icilin vs. DMSO	1 µM icilin vs. DMSO	3 µM icilin vs. DMSO
**22Rv1**	0,8613	0,9791	0,4194
**LNCaP**	0,2372	0,7960	0,1408
**PC-3**	0,7645	0,8475	0,1826
**DU145**	0,3558	0,6788	0,9995

Table S7. Statistical analysis of the effect of AITC, formalin, thymol and cinnamaldehyde on intracellular Ca^{2+} levels in TRPA1-transfected and naive CHO cells. The table shows the statistical data generated by one-way ANOVA followed by Tukey's multiple-comparisons test, n=6/group. *p < 0.001 was considered as statistically significant.

Cell line	100 µM AITC vs. DMSO 5 min	333 µM formalin vs. ECS 30 min	10,000 µM formalin vs. ECS 30 min	100 µM thymol vs. DMSO 7 min	200 µM thymol vs. DMSO 7 min	300 µM cinnam-aldehyde vs. DMSO 5 min	500 µM cinnam-aldehyde vs. DMSO 5 min
**CHO**	0,6139	**0,0022** *	**<0,0001** **		**0,0003** **		**0,0017** *
**TRPA1-CHO**	**<0,0001** **	**0,0032** *		**<0,0001** **		**<0,0001** **	

Table S8. Statistical analysis of the effect of capsaicin on intracellular Ca^{2+} levels in TRPV1-transfected and naive CHO cells. The table shows the statistical data generated by one-way ANOVA followed by Tukey's multiple-comparisons test, n=6/group. *p < 0.001 was considered as statistically significant.

Cell line	0.1 µM capsaicin vs. DMSO 10 min	0.3 µM capsaicin vs. DMSO 10 min	1 µM capsaicin vs. DMSO 10 min
**CHO**			0,3181
**TRPV1-CHO**	**<0,0001** *	**<0,0001** *	**<0,0001** *

Table S9. Statistical analysis of the effect of icilin on intracellular Ca^{2+} levels in TRPM8 expressing and naïve HEK cells. The table shows the statistical data generated by one-way ANOVA followed by Tukey's multiple-comparisons test, n=6/group. *p < 0.001 was considered as statistically significant.

Cell line	1 µM icilin vs. DMSO

	5 min
**HEK**	0,3177
**TRPM8-HEK**	**<0,0001** *

Table S10. Statistical analysis of the effect of AITC on ATP-based viability of prostate adenocarcinoma cell lines. The table shows the statistical data generated by one-way ANOVA followed by Tukey's multiple-comparisons test. *p < 0.01 and **p < 0.001 was considered as statistically significant.

AITC concentration (µM)	22Rv1 sample size (n)	22Rv1: treatment versus CTRL (n=24)	22Rv1: AITC versus antagonist co-treatment	LNCaP sample size (n)	LNCaP: treatment versus CTRL (n=15)	LNCaP: AITC versus antagonist co-treatment
**DMSO**	6	0,1839		6	0,5734	
**1**	6	0,9742		6	>0,9999	
**2**	9	**0,0085** *		6	0,9982	
**10**	6	**<0,0001** **		6	**<0,0001** **	
**50**	18	**<0,0001** **		15	**<0,0001** **	
**50 + A96**	6	**<0,0001** **	0,9722	6	**<0,0001** **	>0,9999
**50 + HC**	6	**<0,0001** **	>0,9999	6	**<0,0001** **	0,9256
**100**	6	**<0,0001** **		6	**<0,0001** **	
**200**	15	**<0,0001** **		15	**<0,0001** **	
**200 + A96**	6	**<0,0001** **	0,9273	6	**<0,0001** **	>0,9999
**200 + HC**	6	**<0,0001** **	0,9785	6	**<0,0001** **	>0,9999

Table S11. Statistical analysis of the effect of AITC on cell counts of prostate adenocarcinoma cell lines. The table shows the statistical data generated by one-way ANOVA followed by Tukey's multiple-comparisons test. *p < 0.01 and **p < 0.001 was considered as statistically significant.

**AITC concentration (µM)**	**22Rv1: treatment vs. CTRL** (n=6)	**LNCaP: treatment vs. CTRL** (n=6)
**DMSO** (n=6)	0,4977	0,4148
**1** (n=6)	**0,0043** *	**0,0012** *
**10** (n=6)	**0,0001** **	**<0,0001** **
**50** (n=6)	**0,0001** **	**<0,0001** **

Table S12. Statistical analysis of the effect of capsaicin on ATP-based viability of prostate adenocarcinoma cell lines. The table shows the statistical data generated by one-way ANOVA followed by Tukey's multiple-comparisons test. *p < 0.05, **p < 0.01 and ***p < 0.001 was considered as statistically significant.

**Capsaicin concentration (µM)**	**22Rv1 sample size**	**22Rv1: treatment vs. CTRL** (n=15)	**22Rv1: capsaicin vs. antagonist co-treatment**	**LNCaP sample size**	**LNCaP: treatment vs. CTRL** (n=12)	**LNCaP: capsaicin vs. antagonist co-treatment**
**DMSO**	12	0,9995		6	>0,9999	
**0.1**	6	0,9998		6	0,1308	
**0.3**	6	0,9196		6	0,1742	
**1**	6	0,9153		6	0,3344	
**5**	6	0,6989		6	**0,0234 ***	
**10**	6	0,9661		6	**0,0028 ****	
**50**	6	0,9214		6	**<0,0001 *****	
**100**	9	**<0,0001 *****		6	**<0,0001 *****	
**100 + SB**	6	**<0,0001 *****	0,2351	6	**<0,0001 *****	0,3283
**200**	6	**<0,0001 *****		6	**<0,0001 *****	

Table S13. Statistical analysis of the effect of capsaicin on the cell counts of 22Rv1 prostate adenocarcinoma cell line. The table shows the statistical data generated by one-way ANOVA followed by Tukey's multiple-comparisons test. *p < 0.01 was considered as statistically significant.

**Capsaicin concentration (μM)**	**22Rv1: treatment vs. CTRL** (n=12)
**DMSO** (n=6)	>0,9999
**0.1** (n=6)	>0,9999
**1** (n=6)	0,9329
**10** (n=6)	0,9281
**100** (n=6)	**0,0016 ***
